# Four Case Reports of Acute Psychosis Secondary to Low Doses of Prednisone/Prednisolone

**DOI:** 10.7759/cureus.20853

**Published:** 2021-12-31

**Authors:** Aquila Lesko, Naciye Kalafat, Maleeha Afreen

**Affiliations:** 1 Psychiatry, Medical College of Wisconsin Green Bay, Green Bay, USA; 2 Psychiatry, Bellin Psychiatric Center, Green Bay, USA; 3 Clinical Sciences, Medical University of Lublin, Lublin, POL

**Keywords:** neuropsychiatric, prednisone, prednisolone, psychosis, steroid-induced

## Abstract

Prednisone, the prodrug of prednisolone, has been implicated as the cause of neuropsychiatric symptoms such as depression, mania, agitation, delirium, dementia, psychosis, and many other affective, behavioral, and cognitive changes. Although the literature suggests that patients on 40 mg or more of prednisone a day are at a greater risk for steroid-induced psychosis, patients on <40 mg are still at risk, and therefore, steroid-induced psychosis should not be excluded from the differential. Prednisone is the prodrug of prednisolone, and the two are comparable on a milligram (mg)-to-mg basis. Here are four case studies, three from the literature and one new, that demonstrate acute psychosis secondary to low-dose prednisone/prednisolone use.

## Introduction

Since the introduction of corticosteroid use in the 1950s, patients presented with psychiatric side effects regularly [1,2]. The neuropsychiatric effects, often misleadingly termed “steroid psychosis” in the historical literature, can present in any combination of affective, behavioral, or cognitive changes within as soon as three days of starting the steroid treatment [3,4]. The neuropsychiatric effects found related to steroid psychosis include depression (35%), mania (31%), agitation, mood lability, anxiety, insomnia, catatonia, depersonalization, delirium (13%), dementia, and psychosis (14%) [3,5]. These percentages represent the most common presentations of steroid psychosis according to a retrospective study by Lewis and Smith [5].

The Boston Collaborative Drug Surveillance Program specifically monitored 718 patients receiving prednisone for adverse reactions and found a strong dose-response relationship for acute psychiatric reactions [6]. Similarly, Lewis and Smith found that there was an increased incidence of psychiatric reactions to steroid treatment with increasing average daily doses of prednisone [5]. Specifically, it has been found that patients receiving daily doses of 40 mg prednisone are at a greater risk of developing steroid psychosis [4]. 

Clinically, these findings may have biased physicians to dismiss steroid psychosis as a differential in cases of neuropsychiatric presentation in the presence of low-dose prednisone. However, cases have demonstrated that doses even as low as 2.5 mg of prednisolone daily have consistently been associated with steroid psychosis without any prior history of mental illness. In this study, we discuss three case reports of patients with findings from the literature on low-dose prednisone-induced psychosis as well as a new case presentation.

## Case presentation

Case one

The first case report describes a 77-year-old man with adrenal insufficiency, Hashimoto’s thyroiditis, and no known psychiatric history [7]. The patient presented to the emergency department (ED) with slow response and multiple leg abrasions from a fall. He was discharged with normal consciousness after receiving cortisone 25 mg in the morning, 12.5 mg in the evening, and thyroxine 50 μg in the morning. Three days later, the patient received a 15-day prescription of prednisolone 10 mg in the morning and 5 mg in the evening. The patient presented to the ED 2.5 months later with hyponatremia (Na = 115 mEq/L), low cortisol, and elevated thyroid-stimulating hormone. He left against medical advice (AMA) after receiving cortisone 50 mg in the morning, cortisone 25 mg, and thyroxine 100 μg in the evening.

Six days later, the patient presented delirious to the ED with a two-day history of insomnia, bizarre speech, and unusual behavior. An adrenal crisis was suspected, so the patient received 100-mg IV hydrocortisone. His delirium did not clear, so additional cortisone of 50 mg was given in the morning and 25 mg in the evening. The patient did not experience resolution of his symptoms and left AMA. The next day, the patient represented to the ED disoriented and agitated with sexual hallucinations, aggression, and self-harming behavior. Acute psychosis was diagnosed, and the corticosteroids were discontinued and olanzapine 5 mg BID was started.

On day 12, his electroencephalography (EEG) showed moderately diffuse encephalopathy, and the patient was started on prednisolone 2.5 mg BID. The patient’s behavior had calmed, but hallucinations persisted. On the 13th day of hospital admission, the patient became agitated, so he was tapered to prednisolone 2.5 mg daily on day 14. On the 15th day of admission, even the low-dose prednisolone was completely removed due to the patient’s hyperactivity, insomnia, and agitation. On day 16, the patient’s psychotic symptoms completely disappeared. The patient was discharged with 100 μg of thyroxine and 5 mg of olanzapine at night.

Case two

A case report of a 48-year-old woman with Sheehan's syndrome describes the neuropsychiatric adverse outcomes of low-dose prednisolone (2.5-10 mg) [8]. The patient was taken to the emergency room showing signs of insomnia, euphoria, and visual hallucinations. She had no previous psychiatric history. The patient was diagnosed with panhypopituitarism secondary to Sheehan’s syndrome and was prescribed prednisolone 10 mg in the morning and 5 mg at night. Following the first dose of prednisolone, the patient became euphoric and experienced insomnia. On day 3, she was anxious and hyperactive. On day 5, the patient experienced agitation, restlessness, and hallucinations. The prednisolone was discontinued on day 7, and she was discharged but was readmitted three days later due to lack of improvement.

A diagnosis of acute psychosis was made, and she was started on lorazepam 4 mg and haloperidol 5 mg. The patient showed rapid improvement from psychosis and thereafter no longer required antipsychotic treatment. Her mental state remained stable for four days. On the fifth day of hospital readmission, a low dose of daily prednisone 2.5 mg was re-administered. The patient immediately showed symptoms of agitation, anxiety, and insomnia once again. The low-dose prednisolone was held, and the symptoms disappeared immediately the following day without the use of antipsychotic drugs. Hydrocortisone was administered in this patient as it directly replaces the much-needed missing hormone for Sheehan’s syndrome. Re-administration of prednisolone with the added hydrocortisone showed improvement to her mental state, and a slow titration of prednisolone to 5.0 mg in the morning over six days while tapering off of hydrocortisone occurred. At the conclusion of the case, the patient was on a daily dose of prednisone 7.5 mg without psychotic symptoms or signs of adrenal insufficiency.

Case three

This case report identified a 21-year-old woman who exhibited symptoms of body, chest, and facial hair [9]. She was diagnosed with hirsutism and had no previous psychiatric history. Her treatment began with low-dose prednisolone 5 mg at night and 2.5 mg in the morning. On day 2, the patient began to display neuropsychiatric symptoms of thought disorder by repeating the phrase “1952.” The patient also reported feeling dazed and increasingly anxious. No pertinent lab results were identified. By day 3, the patient was unable to rest. The patient reported that she could not focus her eyes because her pupils would not constrict and dilate appropriately in the light and dark. The patient also reported clumsiness and misplacing objects. On the fourth day, the patient reported hallucinations and started to again repeat the phrase “1952.” The patient became delusional and reported that she believed someone had hypnotized her. On day 4, the prednisolone was discontinued, and the patient appeared distressed but otherwise free of neuropsychiatric complaints on day 5. 

New case presentation

A 65-year-old woman presented to the inpatient psychiatric unit on emergency detention for confusion, disorientation, and bizarre behavior after medical clearance at the ED. The patient’s basic metabolic panel/complete blood count (BMP/CBC), urine drug testing (UDS), and urine examination (UA) were all found to be normal, and head CT demonstrated no acute findings. The patient presented with a five-day history of new-onset persecutory, jealousy, and bizarre delusions. During the psychiatric evaluation, the patient voiced distress that she was going to die by a suicide bomber on a plane, and she was adamant that her husband was cheating on her with the female police officer that brought her to the hospital. She was admitted on prednisone 2.5 mg daily, hydroxychloroquine 200 mg BID, and sulfasalazine 1,500 mg BID. Chart review revealed at least a six-year history of prednisone use and a desire by the patient's rheumatologist to taper off the medication. On day 1, the patient’s prednisone was discontinued, and olanzapine 2.5 mg BID was started. 

The patient refused all medications except sulfasalazine until day 4, which was when the probable cause with involuntary ordered medication went into effect. On day 3, the patient was found to have a vitamin B12 level of 283 and was given a supplementation of 250 mcg vitamin B12 daily. The patient underwent a Montreal Cognitive Assessment (MOCA) and scored a 16/30 at this time, qualifying for moderate cognitive impairment. Her delusions were maintained, and she was preoccupied with leaving the hospital.

On the morning of day 4, it was noted that the patient’s persecutory, jealousy, and bizarre delusions were beginning to dissolve although the patient had not taken her medication. She no longer believed she would die by a suicide bomber, and she responded “I don’t know” when asked if her husband was cheating. As this was the first day of involuntary medication administration, the patient’s olanzapine was modified to 2.5 mg at night with haloperidol of 2 mg daily IM and benztropine 0.5 mg IM BID as a backup should the patient refuse.

The patient received her first dose of olanzapine 2.5 mg on the night of day 4. On day 5, benztropine 0.5 mg nightly was added because of the patient's concerns of side effects, and a repeat MOCA assessment shows an improved score of 21/30, reducing the previous qualification to mild cognitive impairment. On day 6, olanzapine was increased to 5.0 mg. On day 9, the patient denies the content of all the delusions she had on presentation and expresses an insight into her bizarre behavior being associated with paranoid thoughts. Without the prednisone, the patient begins to experience swelling and pain in the joints, which were well managed with ibuprofen 400 mg every eight hours (q8hrs) PRN. On day 11, it was noted that redirectable residual persecutory delusions were still present when the patient experienced emotional stress. The patient’s olanzapine was increased to 7.5 mg nightly on day 12 to prevent the oscillating nature of the patient’s paranoia. On day 13, the patient’s delusions were found to have completely dissolved, and no residual paranoia was discernible. The patient was discharged home on olanzapine 7.5 mg nightly and benztropine 0.5 mg nightly.

## Discussion

Prednisone is a synthetic glucocorticoid, a prodrug to prednisolone, that enters the nucleus of cells and activates specific nuclear receptors that alters gene expression [10]. It decreases inflammation by decreasing migration of polymorphonuclear leukocytes and reversing capillary permeability and suppresses the immune response by inhibiting proinflammatory cytokine production. It is well known that adverse effects are common in patients receiving glucocorticoids in high doses or over a long time period [10].

While the patient presented here did have a long-term history of prednisone use, the low dose of 2.5 mg daily does not match the typical representation of “steroid psychosis” as doses of 40 mg or higher have been found to pose a much greater risk [4]. However, the patient’s delusions and paranoia were clearly dissolving with no intervention besides discontinuation of her daily 2.5 mg prednisone. After receiving only one dose of 2.5 mg Zyprexa, the patient also saw a five-point improvement on her MOCA assessment. This presentation is consistent with the other cases presented here (cases 2 and 3), in which discontinuation of prednisone/prednisolone saw the resolution of neuropsychiatric symptoms without antipsychotic use.

 It is unlikely the patient was experiencing functional psychosis due to the patient's age and acute onset of delusions without a past psychiatric history. However, the acute onset of delusions that began to resolve solely upon discontinuation of her daily prednisone insinuated an organic psychosis due to glucocorticoid use. While low-dose prednisone is rarely implicated in steroid-induced psychosis, this case series demonstrates that doses as low as 2.5 mg daily have been implicated in causing acute psychosis.

Limitations to this case review include that prednisone and prednisolone were not considered as separate pharmacotherapies. This is because prednisone is a prodrug to prednisolone, only requiring metabolism by the liver to reach glucocorticoid activity, and the two medications are considered comparable on an mg-to-mg basis. Another limitation is that, per the patient’s MOCA scores, she may have a cognitive impairment that can be an organic cause of her psychosis. However, the patient’s improvement of symptoms from steroid discontinuation alone paired with complete resolution of symptoms on discharge makes psychosis due to cognitive impairment more unlikely.

## Conclusions

In conclusion, low-dose prednisone and prednisolone can be implicated in acute psychosis. Evidence shows that discontinuation of low-dose prednisone and prednisolone aided in the cessation of neuropsychiatric symptoms such as delusions, paranoia, hallucination, agitation, and insomnia as described in the case reports of these patients. This is relevant as steroid-induced psychosis may be overlooked as a differential if the dose of prednisone does not match a particular threshold, notably the 40 mg/day standard found by Hall and his colleagues. Appropriate consideration for steroid-induced psychosis, especially when organic psychosis is suspected, should not be ruled out solely based on the dose of prednisone or prednisolone. If steroid-induced psychosis is suspected, discontinuation of the glucocorticoid could be enough to resolve the symptoms.
